# Sympathetic hyperactivity in hypertension via additional intraganglionic cholinergic collateral connections

**DOI:** 10.1113/EP094150

**Published:** 2026-11-11

**Authors:** Minghua Li, Khia Min Sabrina Koh, Susan L. Ingram, Michael C. Andresen, Beth A. Habecker

**Affiliations:** ^1^ Department of Chemical Physiology and Biochemistry Oregon Health and Science University Portland Oregon USA; ^2^ Department of Anesthesiology University of Colorado Anschutz Medical Campus Aurora Colorado USA; ^3^ Department of Medicine Oregon Health and Science University Portland Oregon USA; ^4^ Department of Anesthesiology and Peri‐Operative Medicine Oregon Health and Science University Portland Oregon USA

**Keywords:** angiotensin II, hypertension, patch‐clamp recording, sympathetic ganglion

## Abstract

Sympathetic hyperactivity in hypertension is classically attributed to altered central drive, yet peripheral synaptic mechanisms within autonomic ganglia remain poorly defined. We previously demonstrated excitatory cholinergic collateral synaptic connections between sympathetic ganglion neurons and showed that angiotensin II (AngII)‐induced hypertension increases sympathetic activity in postganglionic neurons independent of the CNS by activating collateral connections. Here, we tested whether AngII‐induced hypertension induces intraganglionic synaptic remodelling in stellate ganglia by quantifying excitatory postsynaptic current (EPSC) latency dynamics, including latency, jitter, response multiplicity and probability of failure. AngII‐induced hypertension increased latency variability of evoked EPSCs, response multiplicity and synaptic failure rates, indicating reduced temporal precision and heterogeneous synaptic transmission. These changes are inconsistent with increased presynaptic release or postsynaptic receptor function and instead suggest altered synaptic circuit organization within stellate ganglia. Collectively, these data provide functional evidence that hypertension is associated with intraganglionic synaptic reorganization consistent with increased circuit complexity. Such peripheral remodelling represents a previously underappreciated mechanism that might contribute to sympathetic overactivity in cardiovascular disease.

## Abstract (highlights)

- **What is the central question of this study?**
  Recent studies revealed that noradrenergic postganglionic sympathetic neurons communicate with each other via collaterals that release acetylcholine. Collateral transmission is increased in explanted sympathetic ganglia after 1 week of angiotensin II‐induced hypertension. Is the enhanced collateral activity attributable to more functional connections between the neurons?
- **What is the main finding and its importance?**
  We stimulated the inferior cardiac nerve retrogradely to activate collaterals selectively and recorded excitatory postsynaptic potentials (EPSPs) in individual neurons. Neurons from hypertensive mice received significantly more EPSPs with greater variability in latency, consistent with increased functional connections within the ganglion. Additional controls ruled out acute effects of angiotensin II on the neurons.

## 1 INTRODUCTION

Sympathetic hyperactivity is a common feature of hypertension (HT) and other cardiovascular diseases, and it contributes to clinical risk (Esler et al., 2001; Floras, 2009; Florea & Cohn, 2014; Grassi et al., 2023; Guyenet, 2006). Sympathetic neurons innervating the heart reside in paravertebral ganglia, where they are activated by release of acetylcholine from preganglionic neurons. Although enhanced central sympathetic mechanisms have been investigated extensively in cardiovascular disease, less attention has been paid to structural and functional changes within peripheral sympathetic ganglia. Classically, ganglionic transmission was viewed as simple monosynaptic connections between preganglionic cholinergic inputs from the spinal cord activating postganglionic noradrenergic neurons that send axons to the heart and other targets. Accumulating evidence indicates that sympathetic ganglia are not simple relay stations but possess complex intrinsic microcircuitry capable of integrating and modulating synaptic input (Clyburn et al., 2022, 2023).

Retrograde labelling shows that the largest population of cardiac neurons are in stellate ganglia (SG), where they are localized predominantly near the inferior cardiac nerve (iCN) (Barrett et al., 2022; Pardini et al., 1989; Rajendran et al., 2019). Previous work identified excitatory cholinergic collateral synaptic connections between SG neurons that could be activated by retrograde stimulation of the iCN (Clyburn et al., 2023). Subsequent studies showed that 1 week of angiotensin II (AngII) infusion not only led to HT, it also increased SG neuronal activity in isolated ganglion explants independent of ongoing CNS input. This suggests that neuron activation was driven in part by changes in an intraganglionic network of cholinergic collaterals (Li et al., 2025). Removal of collateral transmission prevented the increase in neuronal activity, confirming that sympathetic ganglia are not merely passive relays but critical integration nodes that enable amplification or synchronization of postganglionic output (Li et al., 2025).

At least two types of plasticity could account for increased collateral activation of cardiac neurons in explants that lack stimulation from the CNS: (1) increased signalling by existing synapses; or (2) formation or activation of new connections. Both types of plasticity can occur in cardiovascular disease. Sustained HT in Dahl salt‐sensitive rats leads to heart failure and cholinergic transdifferentiation of sympathetic neurons (Kanazawa et al., 2010), and a similar induction of sympathetic ACh production occurs transiently in cardiac sympathetic neurons following myocardial infarction (Olivas et al., 2016). Elevated ACh could result in increased signalling by existing synapses. There is also evidence for potential formation of new synapses in sympathetic ganglia during pathology. The density of cholinergic axons is increased in rabbit SG 1 week after cardiac injury and is accompanied by increased synaptophysin staining (Nguyen et al., 2012). That might reflect sprouting of collateral axons and generation of new synapses, leading to an increased frequency of spontaneous excitatory postsynaptic currents (sEPSCs). Several lines of evidence suggest that synapse formation is the more likely mechanism to increase collateral transmission in AngII‐induced HT. First, the frequency but not the amplitude of sEPSCs is increased after 1 week of AngII HT (Li et al., 2025). Increased ACh release or postsynaptic receptors would be likely to increase the amplitude of responses. Second, we detected no increase in genes associated with cholinergic transmission in SG after either 7 days or 4 weeks of AngII HT (Scalco et al., 2024). Finally, expression of the alpha 7 nicotinic ACh receptor gene is significantly decreased in SG after 1 week of AngII HT (Scalco et al., 2025).

In this study, we tested the hypothesis that AngII HT promotes structural and/or functional reorganization within the SG, leading to enhanced collateral cholinergic synaptic recruitment and increased synaptic complexity (Citri & Malenka, 2008; Magee & Grienberger, 2020). Synaptic latency (the time between stimulus delivery and onset of the synaptic event) or jitter (latency variability) is generally thought to be directly related to the complexity of the synaptic event (Luo et al., 2008; Petreanu et al., 2007). Using whole‐cell voltage‐clamp recordings from SG neurons, we characterize synaptic jitter, latency and the failure rate of evoked excitatory postsynaptic currents excitatory postsynaptic currents (eEPSCs) evoked by retrogradely activating the iCN in saline‐ and AngII‐treated mice.

## 2 MATERIALS AND METHODS

### 2.1 Ethical approval

All experiments and procedures were performed in strict accordance with the *Guide for the Care and Use of Laboratory Animals* as adopted and promulgated by the US National Institutes of Health and approved by the Institutional Animal Care and Use Committee of the Oregon Health & Science University. Procedures adhered to *Experimental Physiology*’s policies regarding animal experiments.

### 2.2 Animals

C57BL/6J mice were used throughout this study. Mice were obtained from Jackson Laboratory and bred and maintained in Oregon Health and Science University's temperature‐ (19°C–21°C) and humidity‐controlled (30%–70%) vivarium with a 12 h light–12 h dark cycle and had *ad libitium* access to normal rodent chow and water. Age‐matched male and female mice 18–25 weeks old were used for all experiments.

### 2.3 Minipump loading and implantation

HT was induced using AngII infusion as previously described (Li et al., 2025). Alzet Mini‐Osmotic Pumps (model 2001) were loaded with either AngII (Worthington Biochemical Corporation 137981, Lakewood, NJ, USA) or saline vehicle, primed for ≥24 h, and placed in a 37°C incubator before implantation into mice according to the manufacturer's instructions. AngII was diluted in 0.9% saline to obtain a dose rate of 700 ng/kg min based on a 25 g mouse.

Mice were anaesthetized with 2% isoflurane in 100% oxygen using a nose cone. Body temperature was monitored through a rectal probe and maintained at 37°C throughout the entire procedure. Hair from the lower neck/scapular region was removed, and a medial incision was made between the scapulae, where the minipump was inserted. The incision was closed with 6–0 silk suture and VetBond. At the end of the procedure, the analgesics buprenorphine (0.1 mg/kg) and meloxicam (5 mg/kg) were administered subcutaneously. Mice were housed individually and monitored daily following surgery until tissue collection 1 week later.

### 2.4 Blood pressure measurements

Mean arterial pressure (MAP) was measured using a non‐invasive tail‐cuff quantification method via a Multichannel Blood Pressure Analysis System (MC4000, Hatteras Instruments). Mice were placed on a heated platform and held in place with specimen holders allowing access to tails, which were threaded through the tail cuff and pulse sensor assembly. The system automatically inflates the tail cuff, measures pulse, systolic and diastolic pressure and calculates MAP and the SD. Measurement sessions consist of 15 inflation and deflation cycles, the first 5 cycles being acclimation cycles and the remaining 10 cycles being for data collection. Mice underwent 2 days of acclimation, when they were placed in the system and went through the inflation–deflation cycles. Pressure measurements were collected on day 5 or 6 after minipump implantation. Systolic, diastolic and MAP data were collected. The MAP in the saline group was 98 ± 12 mmHg (*n* = 7 males and 7 females, mean ± SD). AngII increased MAP significantly: 151 ± 20 mmHg (*n* = 16, 9 males and 7 females, mean ± SD, *P *< 0.001 Student's unpaired *t*‐test).

### 2.5 Stellate ganglion preparation

Intact SG were prepared as described previously (Li et al., 2025). After 7 days of either saline or AngII treatment, mice were deeply anaesthetized with isoflurane and decapitated. Bilateral SG were rapidly dissected and incubated for 90 min at 36°C in constantly oxygenated (95% O_2_ + 5% CO_2_) artificial CSF containing collagenase III (20 mg/mL; Worthington Biochemical Corporation). This enzymatic digestion facilitated the further removal of connective and fatty tissue with fine forceps under a dissection microscope. The isolated ganglion was transferred to a recording chamber perfused continuously (2–3 mL/min) with warm (34°C), carboxygenated artificial CSF ≥30 min before electrophysiological recordings. The bath temperature was maintained and measured by a TC2 bip temperature controller (Cell MicroControls, USA).

### 2.6 Whole‐cell patch‐clamp recordings

SG neurons were visualized using an Olympus BX51W1 light microscope with a 40X water‐immersion objective (Evident Scientific) and visualized on an Olympus DP23M digital monochrome camera (Waltham, MA, USA). Whole‐cell voltage‐clamp recordings were performed using glass micropipettes (2–3 MΩ) filled with potassium gluconate intracellular solution (mM): 138 potassium gluconate, 10 HEPES, 10 KCl, 1 MgCl_2_, 1 EGTA, 0.3 CaCl_2_, 4 MgATP and 3 NaGTP, pH adjusted to 7.3 with KCl, osmolarity 290 mosmol/L. Recording electrodes were pulled from borosilicate glass (1.5 mm in diameter; WPI, Sarasota, FL, USA) on a two‐stage puller (PC‐100, Narishige). Currents were recorded using an Axopatch 200B or a Multiclamp 700B amplifier (Molecular Devices, Sunnyvale, CA, USA). Signals were digitized at 20 kHz, low‐pass filtered at 10 kHz, and acquired using a Digidata 1550B system with pClamp v.11.0 software (Molecular Devices). Series resistance was compensated (<20 MΩ) and monitored continuously during experiments. Unstable recordings were excluded if series resistance or membrane capacitance changed by >15% during the experiment. Each experimental group included successful recordings from at least three or four mice. All neurons were voltage clamped to −60 mV.

### 2.7 eEPSC analysis

The present studies focused exclusively on retrograde activation of efferent axons of SG neurons in the iCN. The iCN was activated using a concentric bipolar electrode (FHC, ME, USA) placed on the visible nerve trunk of the iCN exiting the ganglion. Electrical shocks were brief (100 µs). Once stable intracellular recordings gained access to stellate ganglion neurons, single graded shocks to the iCN typically evoked two responses: one, a short‐latency, large inward current corresponding to a retrogradely conducted action current (rAC); and a much later event, an excitatory postsynaptic inward current (eEPSC). Often these two responses had different intensity thresholds such that they could be recruited separately with the graded shocks. The rAC had the all‐or‐none and early arrival characteristics of the activation of the single axon belonging to recorded SG neurons. eEPSC latency was measured as the time from the stimulus artefact to the onset of the EPSC. Synaptic jitter was measured as the SD of EPSC latency across repeated trials. Low‐jitter, short‐latency EPSCs typically reflect reliable monosynaptic transmission. Increased jitter, multiple EPSC components and higher failure rates suggest recruitment of polysynaptic pathways (Doyle & Andresen, 2001; McDougall et al., 2009).

### 2.8 Spontaneous synaptic events

In a subset of experiments, sEPSCs were recorded. Events were detected using a variable‐amplitude template and threshold set at twice the SD of baseline noise. Events were analysed offline using Clampfit v.11.0 and confirmed visually. sEPSC frequency was calculated over 3–5 min recording epochs following equilibration.

### 2.9 Statistical analysis

Data were analysed using Prism v.11.0 (GraphPad Software Inc., San Diego, CA, USA) and are presented as the mean ± SD. Two‐way ANOVA followed by Tukey's multiple comparison was used for comparisons with more than two groups. The correlation between EPSC jitter and latency was assessed using Spearman correlation analysis. Student's paired *t*‐test was used to compare before and after acute AngII application on sEPSCs. The criterion for significance was set at *P < 0.05.

## 3 RESULTS

### 3.1 AngII‐induced HT enhances sympathetic activity through increased cholinergic collateral synaptic inputs to SG neurons

To determine whether sympathetic hyperactivity in SG neurons from AngII HT mice is driven by increased cholinergic collateral connectivity, we performed retrograde activation of the iCN combined with whole‐cell voltage‐clamp recordings. Threshold stimulation elicited retrograde action currents (rACs), indicating antidromic activation of postganglionic neurons. At higher stimulation intensities, rACs were followed by eEPSCs, suggesting activation of intraganglionic synaptic pathways (Figure 1a).

**FIGURE 1:**
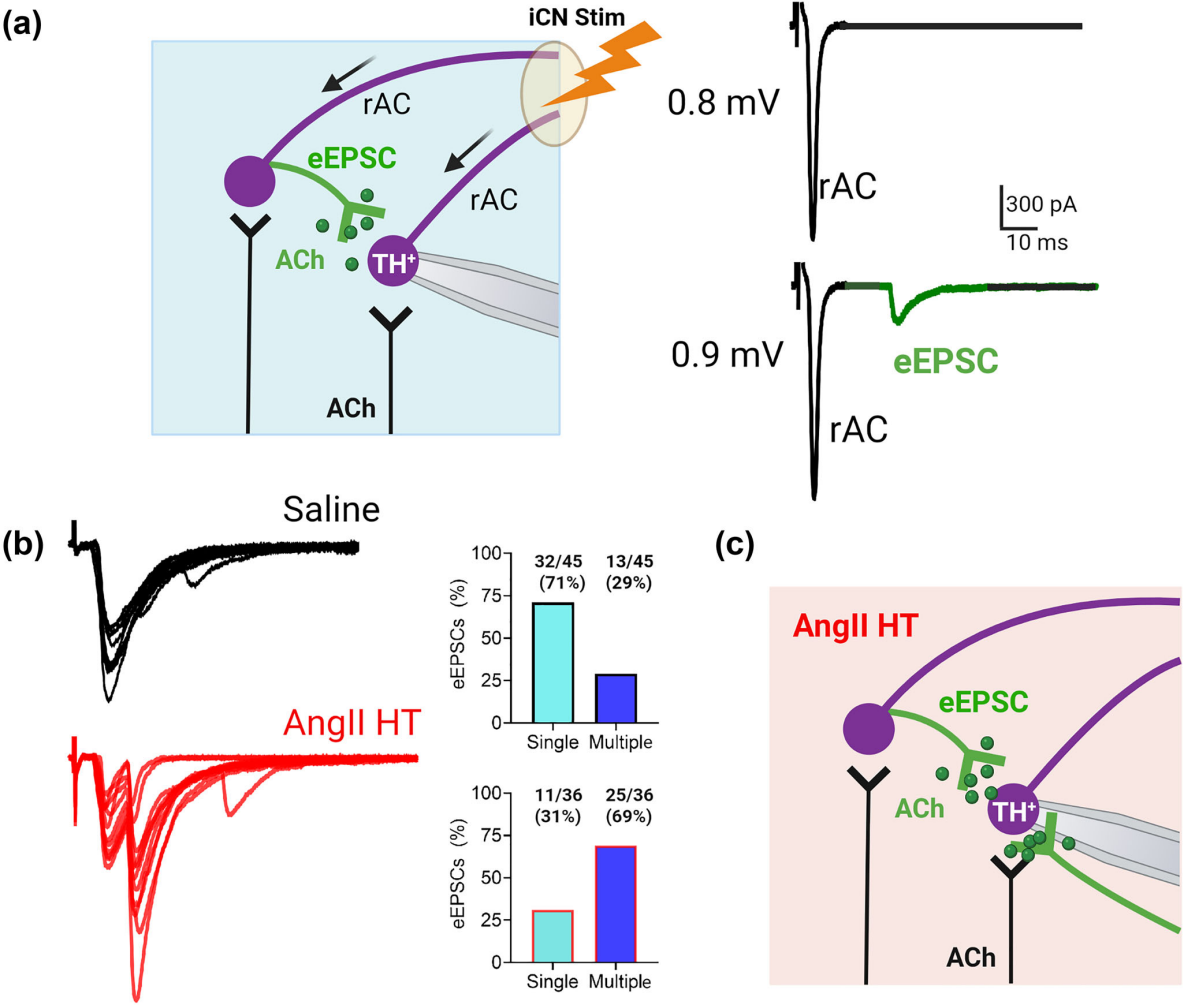
AngII‐induced hypertension increased the proportion of SG neurons receiving multiple EPSCs after retrograde iCN activation. (a) Experimental set‐up. Axons of postganglionic SG neurons expressing tyrosine hydroxylase (TH) were stimulated from the inferior cardiac nerve (iCN). Electrical shocks activate the postganglionic nerve trunk to trigger a retrograde action current (rAC) in a voltage‐clamped SG neuron. Retrograde stimulation can also trigger release of ACh from another SG neuron, resulting in a late‐arriving EPSC. Shocks evoke two distinct events, rAC and EPSCs, that can often be activated separately by graded shock intensities, indicating separate axon thresholds (0.8 vs. 0.9 mA). (b) Representative traces of a high‐intensity shock evoking multiple eEPSCs in a single SG neuron from each treatment group. Bar charts show the quantification of multiple versus single EPSCs in ganglia from saline‐ and AngII‐treated mice. AngII‐treated HT mice display a significantly higher proportion of neurons with multiple eEPSCs compared with saline controls (*P *= 0.0004, Fisher's exact test). There were 13 mice (7 male 6 female) in the AngII HT group and 14 mice (7 male and 7 female) in the saline group. (c) This is consistent with additional collaterals activating the target cell after AngII‐induced HT. Ach, acetylcholine; EPSC, Excitatory postsynaptic current; iCN, inferior cardiac nerve; rAC, retrograde action current, SG, Stellate ganglion; TH, tyrosine hydroxylase.

We next examined whether AngII HT alters synaptic responses in SG neurons. Following a single stimulus, SG neurons from both saline‐treated and HT mice exhibited either single or multiple eEPSCs, suggesting recruitment of additional synaptic inputs. Quantification revealed a marked increase in the proportion of neurons displaying multiple eEPSCs in AngII HT mice compared with saline controls (Figure 1b; *P *= 0.0004, Fisher's exact test). The increased prevalence of multiple EPSCs in AngII‐induced HT mice indicates enhanced synaptic complexity within the SG (Figure 1c).

### 3.2 AngII‐induced HT leads to increased synaptic latency variability in SG neurons

The increased occurrence of multiple EPSCs in AngII HT mice suggests enhanced synaptic complexity within the SG. To determine whether AngII‐induced HT alters synaptic performance characteristics, we analysed the eEPSC latency and the temporal variation of latency (Andresen & Yang, 1995). When multiple presynaptic neurons, such as collaterals, converge onto a single postganglionic neuron, synaptic vesicle release becomes less temporally precise, resulting in increased variability in EPSC latency (synaptic jitter). The SG neurons from hypertensive mice exhibited a significant increase in eEPSC jitter compared with saline controls (Figure 2a). The distribution of jitter was markedly broader in the AngII HT group (0.2–1.9 ms) compared with the relatively restricted range observed in saline controls (0.1–0.6 ms; Figure 2b). Correlation analysis using Spearman's test revealed no significant relationship between jitter and latency in the saline group (Figure 2c). However, there was a significant positive relationship between jitter and latency in the AngII HT group (Figure 2d). Together, these findings indicate that AngII‐induced HT is associated with increased variability in synaptic transmission, probably reflecting the recruitment of additional synaptic inputs to postganglionic neurons within the SG.

**FIGURE 2:**
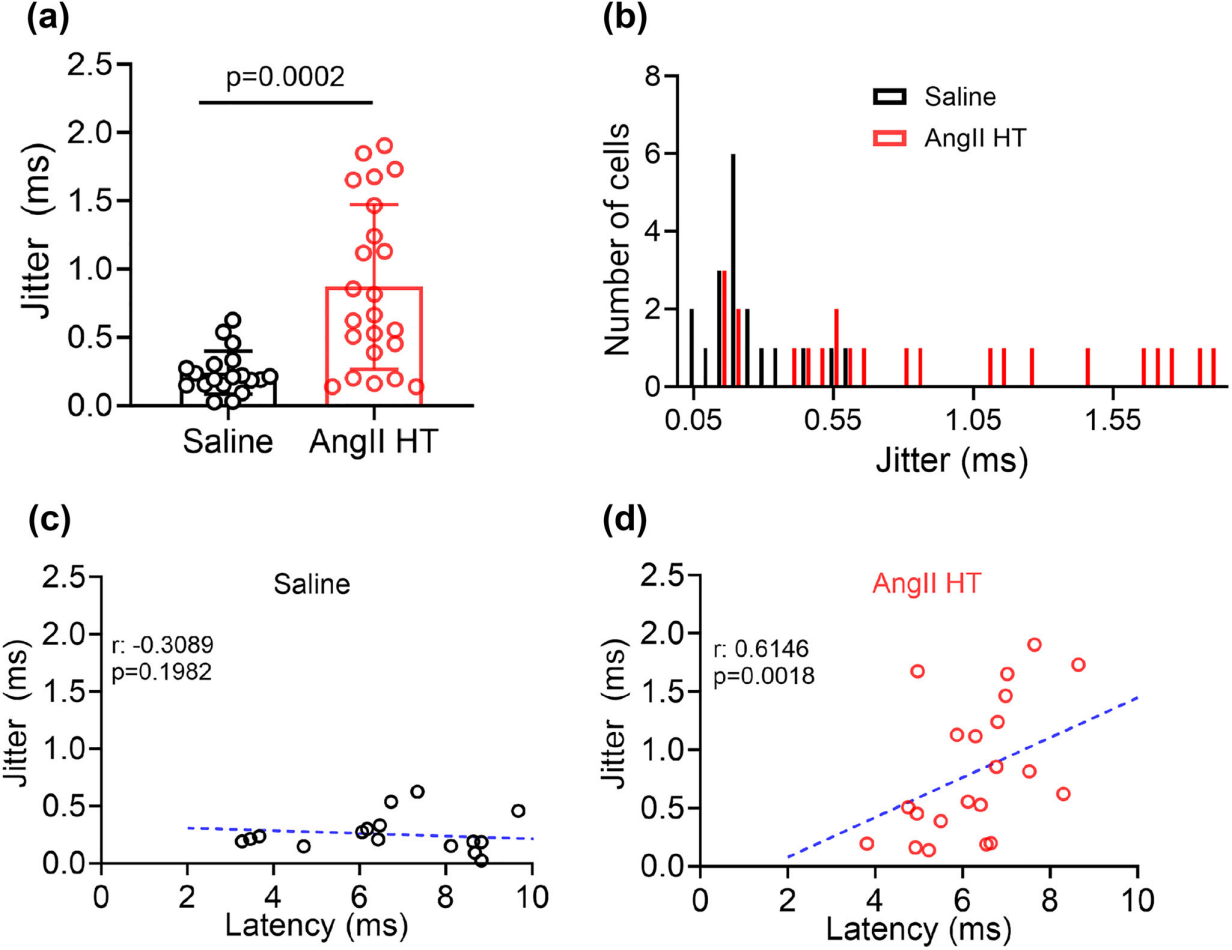
AngII‐induced HT increased eEPSC jitter in SG neurons. All recorded EPSCs evoked by shocks to the iCN are indirect responses that include an action potential triggered in a different SG neuron stimulating ACh release from a cholinergic collateral. The lowest jitter EPSCs are likely to be monosynaptic, but higher jitter responses are likely to be more complex, serial activation of chains of SG neurons. (a) In AngII‐induced hypertensive mice, observed eEPSC jitters increased significantly (0.87 ± 0.60 ms, *n* = 23 cells from 12 mice, 5 male and 7 female) compared with saline controls (0.24 ± 0.15 ms, *n* = 19 cells from 12 mice, 7 male and 5 female; Mann–Whitney *U*‐test, *P *= 0.0002), suggesting an increase in interconnectivity of the intraganglionic collateral networks. (b) In comparison to the saline control, the AngII HT group shows a substantially broader EPSC jitter distribution. (c) Correlation analysis in cells from the saline group revealed no significant relationship between jitter and latency (*r* = −0.3089, *P *= 0.1982, Spearman correlation). (d) In contrast, there was a significant positive relationship between jitter and latency in the AngII HT group (*r* = 0.6146, *P *= 0.0018, Spearman correlation), as expected of lower fidelity EPSCs generated from a more complex chain of spreading collateral connections. Ach, acetylcholine; EPSC, excitatory postsynaptic current; iCN, inferior cardiac nerve; SG, Stellate ganglion.

### 3.3 AngII‐induced HT impairs synaptic reliability through increased eEPSC failures

The increased jitter is likely to reflect engagement of indirect pathways, which are typically associated with greater synaptic unreliability (Bailey et al., 2008; Doyle & Andresen, 2001; McDougall et al., 2009). Therefore, we measured EPSC failure rates using the same stimulation intensity. Intermittent failures of eEPSCs occurred in both saline and AngII HT groups. Quantitative analysis showed that the eEPSC failure rate was significantly higher in AngII‐treated HT mice compared with saline control animals (25% failures in AngII HT vs. 11% in saline, *P *= 0.0154, Mann–Whitney *U*‐test; Figure 3b). The EPSC amplitude was not changed between saline and AngII HT mice (Figure 3c). Furthermore, correlation analysis revealed no relationships between eEPSC failures and amplitude in either saline or AngII HT groups (Figure 3d,e). The high failure rates point to a shift from direct, reliable transmission towards more complex and probabilistic synaptic processing.

**FIGURE 3:**
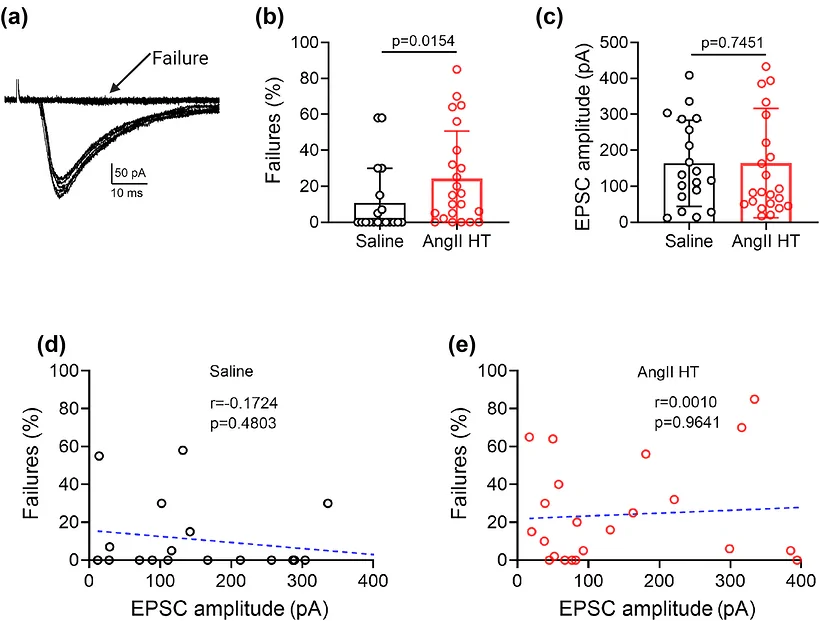
eEPSC failures were higher in cells from AngII‐induced HT mice. (a) Representative eEPSC traces show that sometimes a stimulus shock, even at high intensity, succeeds only intermittently; i.e., failures at identical intensities. (b) Summary data indicate a higher eEPSC failure rate in AngII‐treated hypertensive neurons compared with saline control neurons: 24% (18 of 23 cells; 5 mice, 2 male and 3 female) in AngII vs. 11% (7 of 19 cells; 11 mice, 7 male and 4 female) in saline, Mann–Whitney *U*‐test, *P *= 0.0154). (c) There was no significant difference in EPSC amplitude between saline and AngII‐induced HT (saline, 164 ± 120 pA; AngII HT, 164 ± 152 pA, Mann–Whitney *U*‐test, *P *= 0.7451). (d, e) Correlation analysis revealed no significant relationship between EPSC failures and amplitude in both saline (d; *r* = −0.1724, *P *= 0.4803, Spearman correlation, *n* = 19 cells) and AngII HT groups (e; *r* = 0.0010, *P *= 0.9641, Spearman correlation, *n* = 23 cells). EPSC, excitatory postsynaptic current.

### 3.4 Sympathetic hyperactivity emerges from AngII‐driven hypertensive remodelling, not from acute AngII actions

To determine whether sympathetic hyperactivity in SG neurons results from AngII‐induced HT rather than acute AngII actions, we examined sEPSCs before and during acute AngII perfusion (50 µM; Figure 4a). Acute AngII perfusion did not significantly affect sEPSC frequency or amplitude (Figure 4b,c). These results indicate that short‐term AngII exposure does not modulate spontaneous synaptic transmission in SG neurons, supporting the interpretation that the changes in synaptic performance observed in AngII‐induced HT mice arise from chronic circuit remodelling rather than acute AngII signalling.

**FIGURE 4:**
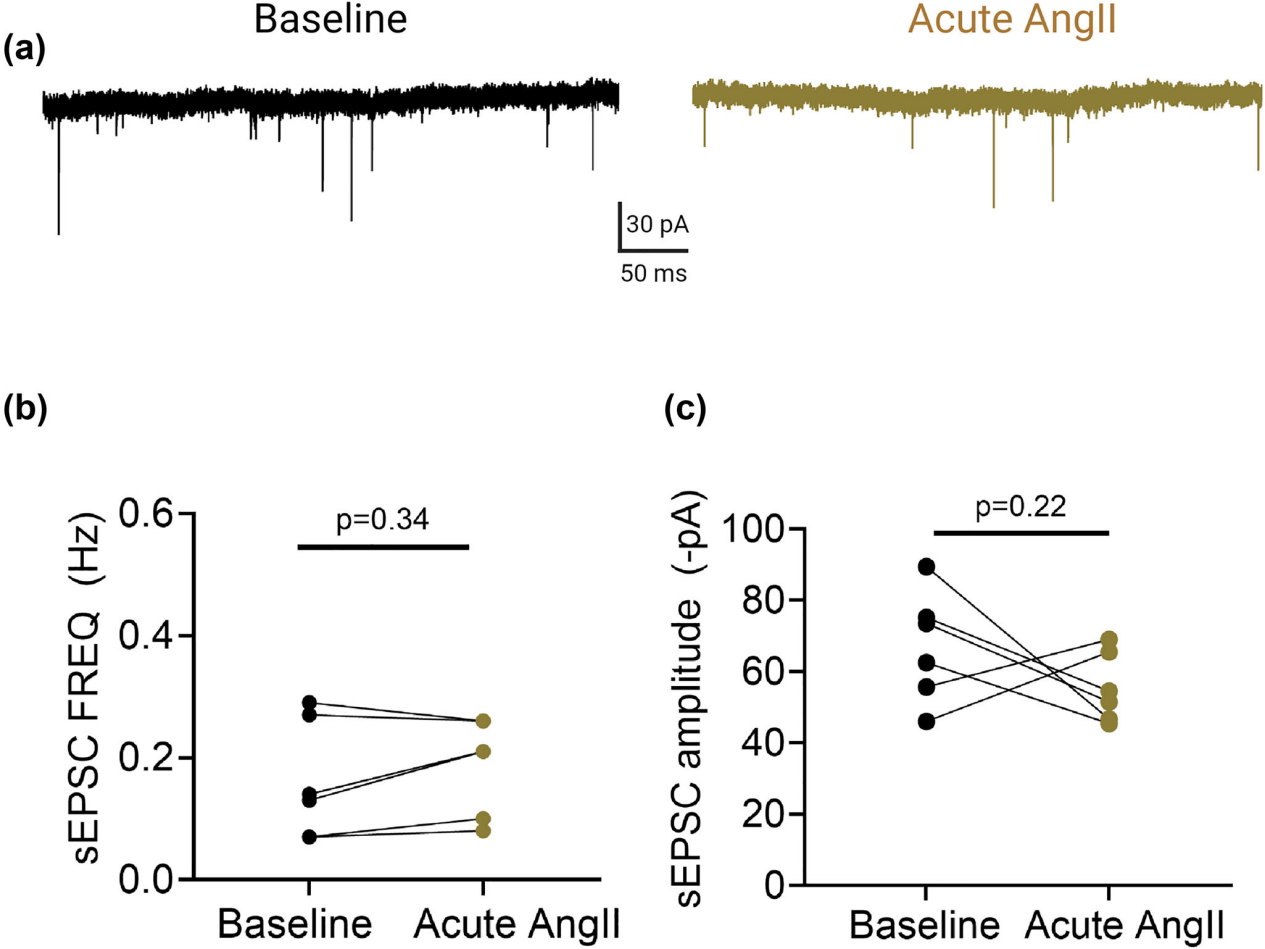
Acute AngII application did not alter spontaneous EPSCs in SG neurons. (a) Spontaneous EPSC traces (sEPSCs) from a single representative SG neuron during baseline conditions and following introduction of AngII (50 µM). (b, c) Quantification of sEPSC frequency (b) and amplitude (c) demonstrated no significant changes following acute AngII application. sEPSC frequency: baseline 0.162 ± 0.10 Hz versus acute AngII 0.187 ± 0.10 Hz (*n* = 6 cells; 4 mice, 2 male and 2 female, Wilcoxon matched‐pairs signed rank test, *P *= 0.34). sEPSC amplitude: baseline, 67.1 ± 15.6 pA versus acute AngII, 55.5 ± 9.8 pA (*n* = 6 cells; 4 mice, 2 male and 2 female, *P *= 0.22).EPSC, excitatory postsynaptic current; SG, Stellate ganglion.

### 3.5 Evoked synaptic timing in SG neurons remains unchanged following acute AngII application

We next examined whether acute AngII application influences the timing of evoked synaptic responses. Neither eEPSC jitter nor latency was altered by acute AngII application (Figure 5a). Additionally, no significant correlation between jitter and latency was observed before or after AngII perfusion (Figure 5b). These results further support that the altered synaptic timing observed in hypertensive mice cannot be explained by acute receptor‐mediated modulation but is likely to reflect chronic circuit remodelling within the SG.

**FIGURE 5:**
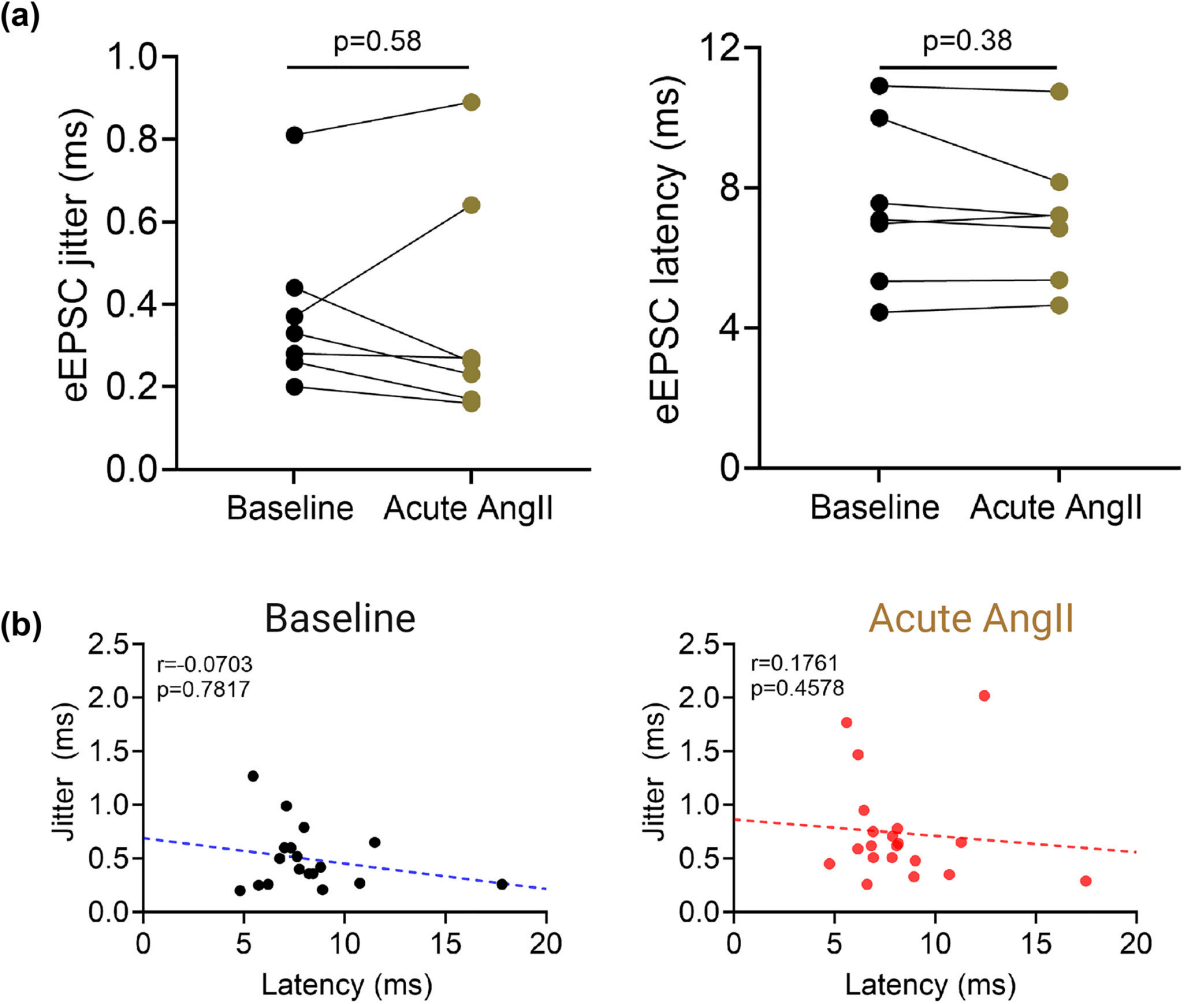
Acute AngII application did not alter retrograde‐evoked EPSCs in SG neurons. (a) Quantification showed no significant change in eEPSC jitter. Baseline, 0.384 ± 0.20 ms versus AngII, 0.374 ± 0.30 ms (*n* = 7 cells; 4 mice, 2 males and 2 females, Wilcoxon matched‐pairs signed rank test, *P *= 0.5781). Latency was also unchanged: baseline, 7.473 ± 2.3 ms versus AngII, 7.163 ± 2.0 ms (*n* = 7 cells; 4 mice, 2 males and 2 females; *P *= 0.3750) following acute AngII application. (b) Correlation analysis shows no significant relationship between eEPSC jitter and latency before or after AngII application. Baseline, *r* = −0.0703, *P *= 0.7817 versus acute AngII, *r* = 0.1761, *P *= 0.4578; Spearman correlation. EPSC, excitatory postsynaptic current; SG, Stellate ganglion

## 4 DISCUSSION

Sympathetic ganglia have commonly been viewed as passive conduits of central autonomic commands, with changes in neuronal activity driven exclusively by increased or decreased activation from the CNS (Jänig, 2008; Végh et al., 2016). However, the presence of collateral cholinergic connections among postganglionic stellate neurons (Clyburn et al., 2023) directly challenges this ‘classical’ framework, which emphasizes simple, monosynaptic synaptic transmission from the CNS to the periphery. These axon collaterals are well positioned to form local amplification loops within the ganglia. Consistent with this concept, our recent work demonstrates that AngII‐induced HT increases sympathetic neuron activity independent of central inputs (Li et al., 2025). In the present study, we investigated the underlying mechanisms of this increased neuronal activity by interrogating synaptic timing metrics, including EPSC latency, jitter and failure rates (Andresen & Yang, 1990; Kasparov & Paton, 1999; Sabatini & Regehr, 1996, 1999). The combined increases in EPSC latency variability (jitter), response multiplicity and failure probability provide strong functional evidence that AngII‐induced HT drives synaptic reorganization within the SG. Importantly, these changes indicate enhanced intraganglionic circuit complexity (addition of new synapses) rather than an increase in synaptic strength owing to enhanced neurotransmitter release or increased postsynaptic receptor expression.

Sympathetic postganglionic neurons have a single axon exiting the ganglion to reach a target tissue. To evaluate intraganglionic interconnections selectively, we activated stellate postganglionic neurons by applying electrical shocks to iCN axons, a method we used to identify collaterals in the past (Clyburn et al., 2023). These shocks activate retrogradely conducted action potentials that are recorded as somatic action currents (rACs) in voltage‐clamped stellate ganglion neurons. The rACs arrive with little delay and low jitter and are unaffected by the nicotinic antagonist hexamethonium (HEX) because they reflect activation of voltage‐gated sodium channels. In many SG neurons, these iCN shocks also evoke late‐arriving synaptic currents (EPSCs) that are blocked by HEX (Clyburn et al., 2023). All these eEPSCs involve at least two SG neurons: the neuron that is retrogradely activated to stimulate release of ACh in the ganglion, and the neuron where we record the eEPSC. Our new data indicate that in control ganglia iCN stimulation elicits a single EPSC in most neurons, with a wide range of latencies but little variability in latency across repeated stimulations. This consistency suggests that the range of latencies across cells is generated by different distances between the stimulated and recorded neurons. In contrast, 1 week of AngII‐induced HT profoundly increased the average jitter of eEPSCs but did not alter rAC characteristics. The broader variability of latency values indicates that AngII‐induced HT increased the complexity of collateral synaptic connections without altering basic synaptic or action potential mechanisms. This greater complexity of ganglionic interconnections is evident in the higher incidence of multiple EPSC complexes and high failure rates of retrogradely eEPSCs. The correlation between greater latencies and higher jitter suggests longer, more complex transit paths within the ganglion, reflecting augmented multi‐step intraganglionic communication. An increase in the complexity of intraganglionic connections increases the likelihood of failures in triggering retrogradely eEPSCs, and we observed significantly greater failures of eEPSCs in AngII HT, providing independent evidence of greater circuit complexity.

Synaptic plasticity occurs at multiple levels. Functional plasticity involves modification of neurotransmitter release, receptor expression, and signalling pathways at the molecular level, whereas structural plasticity reorganizes connectivity that alters signal routing and temporal integration (Abraham et al., 2019). The observed increases in EPSC jitter, multiple response components, elevated failure rates and the positive correlation between latency and jitter are more consistent with connectivity‐based plasticity than with changes in synaptic transmission. The lack of significant changes in eEPSC amplitude between saline and AngII HT groups here and in our previous study (Li et al., 2025) also supports this interpretation.

The mechanisms that underlie increased cholinergic connections within SG during AngII‐induced HT are not understood. Although CNS inputs are missing in our explant studies, increased CNS activation or infiltration of immune cells into the ganglion during the 1 week of HT might contribute to remodelling, and culture studies suggest that glia might be involved. Sympathetic neurons generate cholinergic synapses with other neurons in culture, even as they form noradrenergic synapses with target cells, such as cardiac myocytes (Furshpan et al., 1986). This is similar to the cholinergic collaterals observed in vivo. Addition of satellite glial cells to cultured sympathetic neurons increased the spontaneous activity of the neurons (Enes et al., 2020). Electrophysiological studies indicate that the glial cells do not alter the intrinsic excitability of the neurons but instead enhance cholinergic synapse formation in the culture (Enes et al., 2020). This raises the possibility that glia cells release factors in the context of AngII‐induced HT that contribute to circuit remodelling within the ganglion. Additional studies will be needed to identify those mechanisms.

AngII exerts effects across multiple tissues, including central neurons, where it stimulates sympathetic outflow (Veerasingham & Raizada, 2003; Zhang et al., 1999), the heart (Mello & Danser, 2000) and peripheral sympathetic neurons, where it increases noradrenaline production (Boadle et al., 1969). Furthermore, sympathetic ganglia in both rodents and humans express all components of the renin–angiotensin system (Bardsley et al., 2019). To distinguish between the effects of chronic AngII‐induced HT and direct receptor‐mediated modulation, we examined the impact of acute AngII application in SG neurons and detected no change in activity. The lack of acute effects argues against rapid receptor‐mediated changes in synaptic transmission or postsynaptic responsiveness, supporting the idea that the enhanced synaptic connectivity arises from longer‐term, HT‐driven remodelling. Such structural adaptations might persist even after acute fluctuation in AngII levels, contributing to sustained sympathetic overactivity (Chrysant, 1998; Kurdi & Booz, 2011; Xue & Johnson, 2023).

Sympathetic hyperactivity is a hallmark of many cardiovascular diseases. Increased CNS‐derived sympathetic drive contributes significantly to this dysregulation (van Weperen & Vaseghi, 2026), but targeting the CNS to lower sympathetic activation has not proved effective in patients (Cohn et al., 2003), whereas targeting peripheral sympathetic transmission with drugs (Lopez‐Sendon et al., 2004) or sympathetic ganglionectomy (Vaseghi et al., 2017) provides clear clinical benefits (Herring et al., 2019, 2025). Our previous finding showed that sympathetic neurons remain hyperactive even after removal of central inputs (Li et al., 2025), and new data indicate that sympathetic overactivity persists in humans with HT even in the presence of medication to lower blood pressure (Grassi et al., 2025). Building on this, the present study demonstrates that chronic AngII‐induced HT does not merely elevate sympathetic activity within the stellate ganglion; it also drives reorganization of synaptic circuit architecture within the stellate ganglion. This increased circuit complexity among ganglion neurons might amplify cardiac sympathetic output, thereby contributing to cardiovascular pathology.

## 5 CONCLUSION

By identifying sympathetic ganglia as dynamic substrates of hypertensive remodelling, this work broadens the mechanistic framework of neurocardiac dysregulation. In particular, it highlights peripheral synaptic reorganization as a potential driver of sympathetic hyperactivity. These findings suggest a novel therapeutic target that extends beyond traditional central and cardiac approaches.

## AUTHOR CONTRIBUTIONS

All authors contributed to the conception or design of the work or to data acquisition and analysis or to interpretation. All authors contributed to drafting the work or revising it for intellectual content. In addition, all authors approved the final approved the final version of the manuscript and agree to be accountable for all aspects of the work in ensuring that questions related to the accuracy or integrity of any part of the work are appropriately investigated and resolved. All persons designated as authors qualify for authorship, and all those who qualify for authorship are listed.

## CONFLICT OF INTEREST

The authors declare no conflicts of interest.

## GENERATIVE AI STATEMENT

No generative AI tools were used in generating data or writing the manuscript.

## Data Availability

All data supporting the results described here are presented in the paper.
